# Supplementary material for: Splice-Junction-Based Mapping of Alternative Isoforms in the Human Proteome
Source: Cell Rep. Author manuscript; Available in PMC 2020 Jan 15. (PMC6961840; doi:10.1016/j.celrep.2019.11.026)
Supplement: 3 [file NIHMS1546469-supplement-3.zip › DF2/PXD006675/PulmonaryVein-151-P56159-VVPFISVEHIPK.pdf]

A

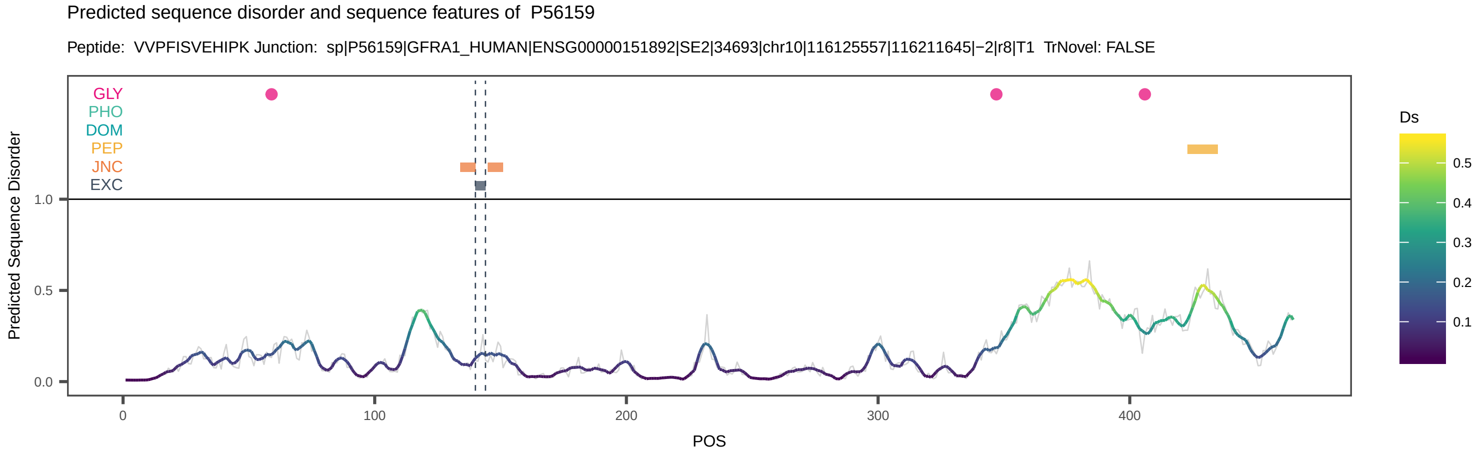
Predicted sequence disorder and sequence features of P56159
Peptide: VVPFISVEHIPK Junction: sp|P56159|GFRA1_HUMAN|ENSG00000151892|SE2|34693|chr10|116125557|116211645|−2|r8|T1 TrNovel: FALSE
GLY
PHO
DOM
PEP
JNC
EXC
Predicted Sequence Disorder
1.0
0.5
0.0
0
100
200
300
400
POS
Ds
0.5
0.4
0.3
0.2
0.1

B

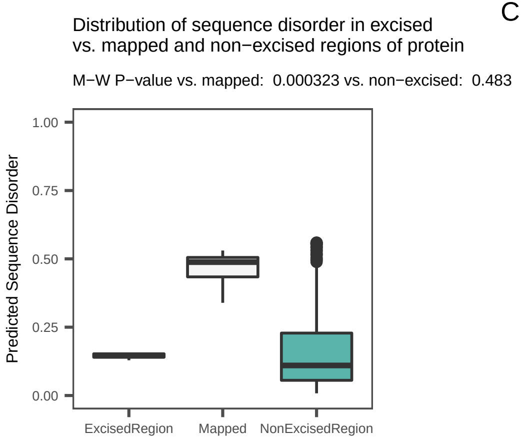
Distribution of sequence disorder in excised
vs. mapped and non−excised regions of protein
M−W P−value vs. mapped: 0.000323 vs. non−excised: 0.483
Predicted Sequence Disorder
1.00
0.75
0.50
0.25
0.00
ExcisedRegion
Mapped
NonExcisedRegion

C
